# Design, Synthesis and Anticancer Activity of New Polycyclic: Imidazole, Thiazine, Oxathiine, Pyrrolo-Quinoxaline and Thienotriazolopyrimidine Derivatives

**DOI:** 10.3390/molecules26072031

**Published:** 2021-04-02

**Authors:** Ameen Ali Abu-Hashem, Sami A. Al-Hussain, Magdi E. A. Zaki

**Affiliations:** 1 Heterocyclic Unit, National Research Centre, Photochemistry Department, Dokki, Giza 12622, Egypt; mezaki@imamu.edu.sa or; 2 Chemistry Department, Faculty of Science, Jazan University, Jazan 45142, Saudi Arabia; 3 Department of Chemistry, Faculty of Science, Imam Mohammad Ibn Saud Islamic University (IMSIU), Riyadh 13318, Saudi Arabia; sahussain@imamu.edu.sa

**Keywords:** thienopyrimidinone, thienotriazolopyrimidinone, 1,2,4-triazole, thiazine, pyrrole, oxathiinoquinoxaline, imidazole, imidazopyrrolotriazole, anticancer activity

## Abstract

In this article, we showed the synthesis of new polycyclic aromatic compounds, such as thienotriazolopyrimidinones, *N*-(thienotriazolopyrimidine) acetamide, 2-mercapto-thienotriazolo-pyrimidinones, 2-(((thieno-triazolopyrimidine) methyl) thio) thieno-triazolopyrimidines, thieno-pyrimidotriazolo-thiazines, pyrrolo-triazolo-thienopyrimidines, thienopyrimido-triazolopyrrolo-quinoxalines, thienopyrimido-triazolo-pyrrolo-oxathiino-quinoxalinones, 1,4-oxathiino-pyrrolo- triazolothienopyrimidinones, imidazopyrrolotriazolothienopyrimidines and 1,2,4-triazoloimidazo- pyrrolotriazolothienopyrimidindiones, based on the starting material 2,3-diamino-6-benzoyl-5- methylthieno[2,3-d]pyrimidin-4(3*H*)-one (**3**). The chemical structures were confirmed using many spectroscopic ways (IR, ^1^H, ^13^C, −NMR and MS) and elemental analyses. A series of thiazine, imidazole, pyrrole, thienotriazolopyrimidine derivatives were synthesized and evaluated for their antiproliferative activity against four human cancer cell lines, i.e., **CNE2** (nasopharyngeal), **KB** (oral), **MCF-7** (breast) and **MGC-803** (gastric) carcinoma cells. The compounds **20**, **19**, **17**, **16** and **11** showed significant cytotoxicity against types of human cancer cell lines.

## 1. Introduction

**Figure 1:**
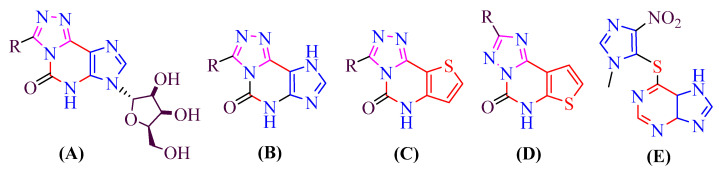
Chemical structures of formerly synthesized drugs: triazolopurines and thienotriazolo- pyrimidines analogues.

Cancer is one of the most recent serious diseases that afflict humans and ultimately leads to their death. From here, researchers began to develop and discover much effective anticancer therapeutics, such as treatment of cancer using chemotherapy [1] and heterocyclic compounds, such as thienopyrimidine, triazolopyrimidine, quinoxaline and imidazole derivatives containing anticancer drugs [2], as follows: Azathioprine, Pimonidazole, Dacarbazine, Misonidazole, Fadrozole, Tipifarnib, Bendamustine, Indimitecan and Nilotinib. Therefore, the chemical studies of each of thiophene, pyrimidine, triazole, thiazine, pyrrole, quinoxaline and imidazole nucleus play a significant role in the synthesis of a diversity of fused heterocyclic compounds having a wide range of biological and pharmacological activities. Accordingly, previous scientific studies confirmed that thienopyrimidine derivatives have various biological and pharmacological activities, such as antibiotics [3], antimicrobial [4,5], anticonvulsants [6], antiviral [7], antioxidant and antitumor agents [8], anticancer [9] and mitotic arrest of breast cancer [10], anti-inflammatory and analgesic activities [11,12], antiglaucoma agents [13], platelet aggregation inhibitors [14], anti-hyperlipidemia [15], antidepressant, anti-inflammatory and antimicrobial activities [16]. In addition, the thiazolopyrimidine, 1,2,4-triazolopy- rimidine and thienotriazolopyrimidinone derivatives possess such biological activities as anti-inflammatory and analgesic activity [17,18], antimicrobial [19] and anticancer activity through a potential of the enzyme (PARP-1) inhibition [20]. Lately, in connection to continuing work in the synthesis and biological evaluation of new polycyclic fused thienopyrimidine, purine and 1,2,4-triazole systems, purine derivatives are of great importance and wide applications in many biological activities, such as antitumor [21] and the potential xanthine oxidase (XO)-inhibitory activities, as well as many biological activities when fused with 1,2,4-triazole ring, shown in Figure 1, as the following: 7β-D-ribofuranosyl-1,2,4-triazolopurines (A) [22], 1,2,4-triazolopurines (B) [23], thienotriazolopyrimidinones (C) and 2-sub-thienotriazolopyrimidinones (D) as a new class of the XO inhibitors [24]. Additionally, Azathioprine (E) is an anticancer drug that possesses considerable potential, because of its ability to interfere with DNA prepared and then stop growth and division cells and it is used for the treatment of metastatic malignant melanoma and cell carcinoma of the pancreas [25].

**Figure 2:**
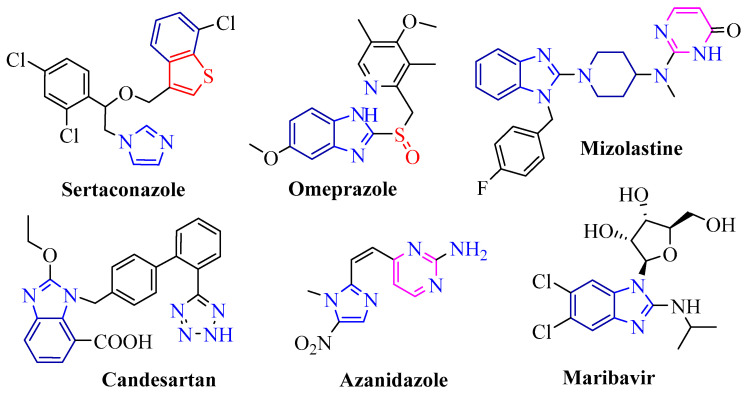
Chemical structures of formerly synthesized drugs: benzimidazoles and imidazoles.

The nitrogen atoms containing heterocycles, especially, display a different range of biological activities, due to their similarities with numerous synthetic and natural molecules with recognized biological activities [26]. The benzimidazole and imidazole rings have been generally used as the substantial basic structure for the development of therapeutic molecules of biological and pharmaceutical activities. An example of five-membered heterocycles is imidazole, which is spread between the significant biological building blocks. Thus, many drugs contain imidazole, such as the following: Sertaconazole is used as an antifungal agent [27]. Omeprazole is antiulcer and controls the acid secretion in the stomach and it is considered clinically superior to H_2_-receptor antagonists [28,29]. Mizolastine is an antihistaminic and potent antagonist at H_1_ receptor sites for the treatment of allergic rhinoconjunctivitis and urticarial [30]. Candesartan is used as a receptor antagonist, because it contains a bulky lipophilic group, carboxylic acid, the biphenyl group that is more efficient than the tetrazole analogue [31]. Azanidazole is an antiparasitic drug and used as an antibacterial and antiprotozoal drug [32]. Maribavir is an antiviral drug, which is used in the treatment and prevention of human cytomegalovirus (HCMV) disease in hematopoietic stem cell/bone marrow transplant patients [33], as shown in Figure 2.

This article is a supplement of our former work about the preparation of heterocyclic compounds resulting from pyrimidine and thienopyrimidine derivatives, which possess biological activities [4,5,8,11,12,19,20,21,34,35,36,37,38,39,40,41,42,43,44,45,46,47,48]. In this manuscript, these compounds, thienotriazolopyrimidinone, thienopyrimidotriazolopyrrolooxathiinoquinoxalines and 1,2,4-triazoloimidazopyrrolotriazolothienopyrimidinedione derivatives were synthesized from 2,3-diamino-6-benzoyl-5-methylthieno[2,3-d]pyrimidinone and evaluated as having antiproliferative activities against types of human carcinoma cancer cell lines.

## 2. Results and Discussion

### 2.1. Synthesis

**Scheme 1:**
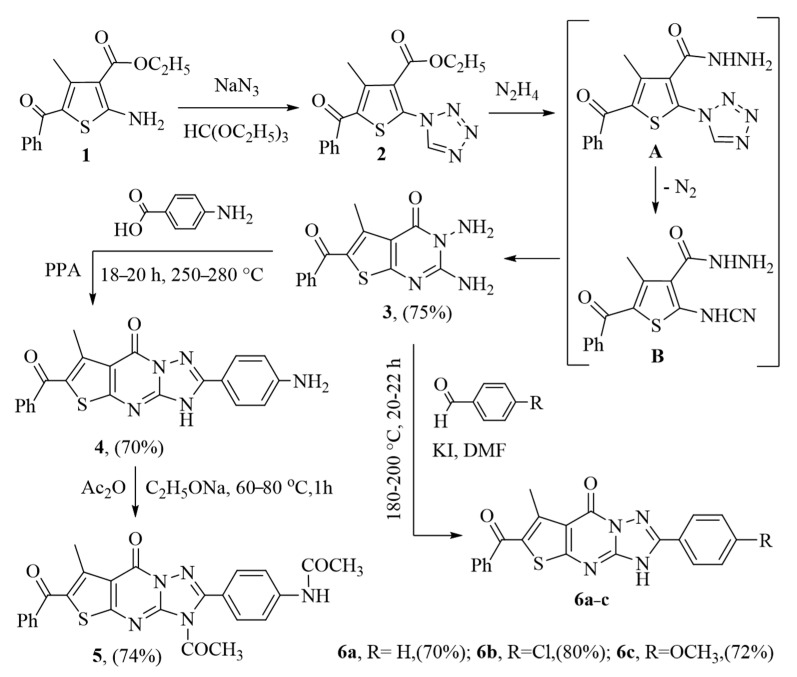
Synthesis of 2-(sub)-thieno[2,3-d][1,2,4]triazolopyrimidinone derivatives.

The 2-(4-aminophenyl)-6-benzoyl-7-methylthieno[2,3-d][1,2,4]triazolo[1,5-a] pyrimidin-8(3*H*)-one (**4**) was synthesized via the oxidative cyclization of 2,3-diamino- 6-benzoyl-5-methylthieno[2,3-d]pyrimidin-4(3*H*)-one (**3**) [4,18] and 4-aminobenzoic acid in polyphosphoric acid at 250–280 °C for 18–20 h. The IR spectra of compound **4** showed the presence of a broad bands absorption at 3405 and 3355 cm^−1^ of one NH_2_ and one NH group and two bands absorption at 1680 and 1665 cm^−1^ for two carbonyl groups. The proton nuclear magnetic resonance (^1^H–NMR) spectrum of compound **4** displayed two singlet broad signals at *δ* 6.10 and 10.70 ppm, agreeing to the three protons of the one NH_2_ and one NH group (D_2_O-exchangeable). The reaction of the latter compound **4** with sodium ethoxide (NaOEt) afforded the conforming sodium salt, which, upon treatment with acetic anhydride (Ac_2_O), provided the acetylated (**5**). In another way, the acetylated of compound **4** with acetic anhydride, itself, for one hour formed the *N*-(4-(3-acetyl- 6-benzoyl-7-methyl-8-oxo-3,8-dihydrothieno[2,3-d][1,2,4]triazolo[1,5-a]pyrimidin-2-yl) phenyl) acetamide (**5**) in 74% yield. The ^1^H–NMR spectrum of compound **5** demonstrated one singlet broad signal at 11.90 ppm, correspondent to the one proton of the one NH group with D_2_O-exchangeable. Moreover, refluxing 2,3-diamino-benzoyl-methylthieno- pyrimidinone (**3**) with a proper aromatic aldehyde, namely benzaldehyde, 4-chloro- benzaldehyde or 4-methoxybenzaldehyde, respectively, in dimethylformamide solution, having a catalyst amount of potassium iodide for an extended time, gave the products 6-benzoyl-2-(4-substituted-phenyl)-7-methylthieno[2,3-d][1,2,4]triazolo[1,5-a]pyrimidin- 8 (3*H*)-one (**6a**–**c**) in good yields. The ^1^H–NMR spectrum of compound **6a** established one singlet broad signal at *δ* 10.55 ppm, corresponding to the one proton of NH group with D_2_O-exchangeable. The mass spectra of compounds **6a**–**c** showed molecular ion peaks at m/z 386 (M^+^, 90%), 420 (M^+^, 100%) and 416 (M^+^, 95%), respectively, as shown in Scheme 1.

**Scheme 2:**
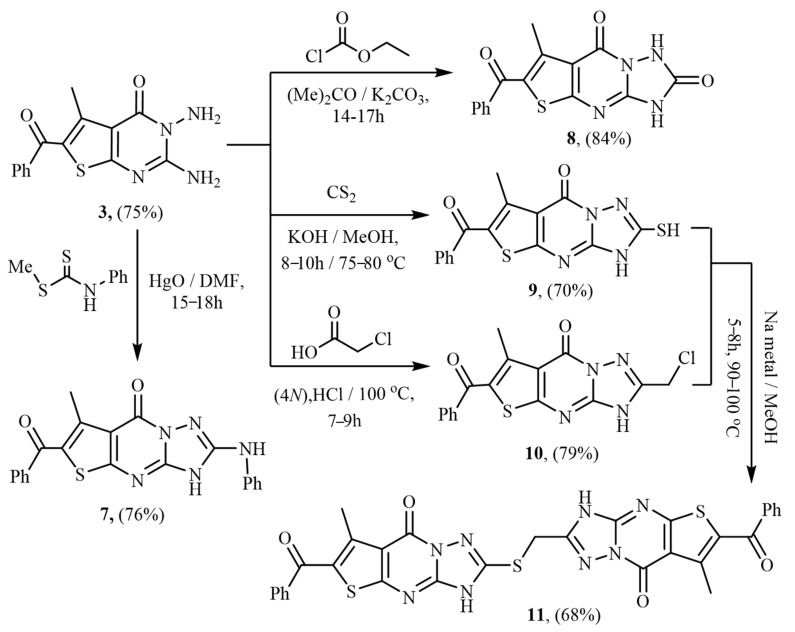
Synthesis of methylthieno[2,3-d][1,2,4]triazolo[1,5-a]pyrimidinone derivatives.

Heating of 2,3-diamino-benzoyl-methylthienopyrimidinone (**3**) with methylphenyl- carbamodithioate and red mercury (II) oxide (HgO) catalyzed in dimethylformamide to give 6-benzoyl-7-methyl-2-(phenylamino)thieno[2,3-d][1,2,4]triazolo[1,5-a]pyrimidin-8 (3*H*)-one (**7**) in good yield. IR spectrum of compound **7** revealed the presence of broad bands absorption at 3360 and 3320 cm^−1^ of (2NH) groups and 1715 and 1676 cm^−1^ of two carbonyl groups, and ^1^H–NMR spectrum of compound **7** displayed two singlet broad signals at *δ* 9.60 and 10.80 ppm, conforming to the two protons of the (2NH) groups with D_2_O-exchangeable. Moreover, 6-benzoyl-7-methylthieno[2,3-*d*][1,2,4]triazolo[1,5-a]pyri- midine-2,8(1*H*,3*H*)-dione (**8**) was prepared by reacting of 2,3-diamino-benzoyl- methyl- thienopyrimidinone (**3**) with ethyl-chloroformate and anhydrous potassium carbonate in dry acetone. IR spectra of compound **8** showed the absence of some absorption bands for two amino groups, with the presence of bands absorption at 3352 and 3310 cm^−1^ of (2NH) groups and 1722, 1685 1674 cm^−1^ of three carbonyl groups. The ^1^H–NMR spectrum of compound **8** designated two singlet broad signals at *δ* 10.20 and 11.10 ppm, corresponding to the two protons of the (2NH) groups. On the other hand, heating and stirring of 2,3-diamino-benzoyl-methylthienopyrimidinone (**3**) with carbon disulfide in an methanolic potassium hydroxide solution led to the formation of 6-benzoyl-2-mercapto-7-methylthieno[2,3-d][1,2,4]triazolo[1,5-a]pyrimidin-8(3*H*)-one (**9**). IR spectra of compound **9** presented the presence of broad bands absorption at 3345 and 2372 cm^−1^ of (NH) and (SH) groups, respectively, and ^1^H–NMR spectrum of compound **9** revealed two singlet signals at 2.62 and 9.05 ppm, agreeing to two protons of the (SH) and (NH) groups with D_2_O-exchangeable, respectively. In addition, the reaction of 2,3-diamino-benzoyl-methylthienopyrimidinone (**3**) with chloroacetic acid in hydrochloric acid (4*N*, HCl) produced the 6-benzoyl-2-(chloromethyl)-7-methylthieno[2,3-d][1,2,4]triazolo[1, 5-a] pyrimidin-8 (3*H*)-one (**10**). IR spectra of compound (**10**) obtained the presence of bands absorption at 3341 cm^−1^ of (NH) and 1724 and 1680 cm^−1^ of two carbonyl groups, and ^1^H–NMR spectrum of compound **10** discovered one singlet signal at 9.15 ppm, conforming to the one proton of the (NH) group (D_2_O-exchangeable). Thus, the heating under reflux of 2-mercapto-thienotriazolopyrimidinone (**9**) with 2-(chloromethyl)-thienotriazolopyrimi- dinone (**10**) in an methanolic sodium metal solution produced the 6-benzoyl-2-(((6-ben- zoyl-7-methyl-8-oxo-3,8-dihydrothieno[2,3-d][1,2,4]triazolo[1,5-a]pyrimidin-2-yl)methyl)thio)-7-methylthieno[2,3-d][1,2,4]triazolo[1,5-a]pyrimidin-8(3*H*)-one (**11**). The ^1^H–NMR spectra of compound **11** exhibited two broad singlet signals at *δ* 9.80 and 10.10 ppm, identical to the two protons of the (2NH) groups (D_2_O-exchangeable). Mass spectra of compounds (**7**- **11**) showed molecular ion peaks at m/z 401 (M^+^, 95%), 326 (M^+^, 100%), 342 (M^+^, 93%), 358 (M^+^, 91%) and 664 (M^+^, 90%), respectively as shown in Scheme 2.

**Scheme 3:**
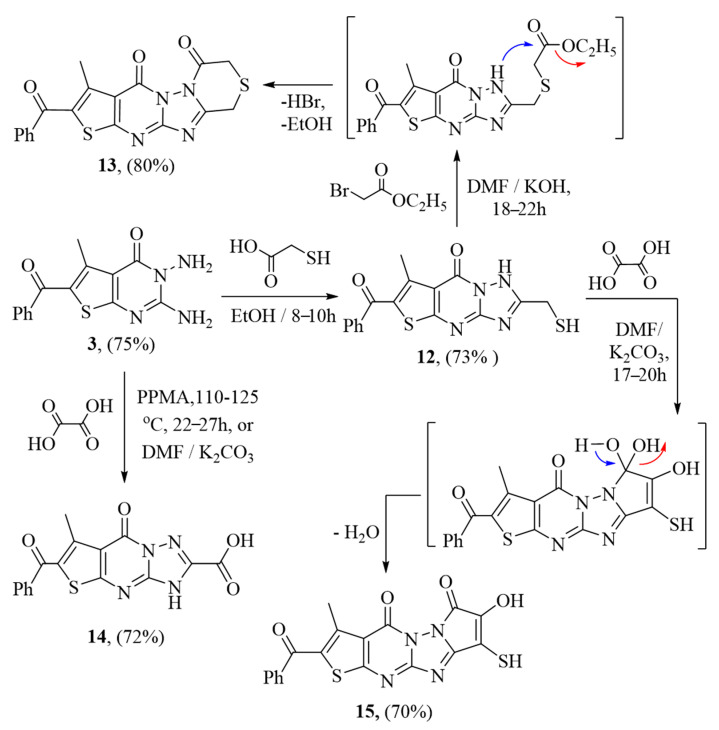
Synthesis of thiazine, pyrrolo-1,2,4-triazolo-thieno[2,3-d]pyrimidinones.

In addition, 6-benzoyl-2-(mercaptomethyl)-7-methylthieno[2,3-d][1,2,4]triazolo[1,5-a]pyrimidin-8 (1*H*)-one (**12**) was synthesized from 2,3-diamino-benzoyl-methylthieno- pyrimidinone (**3**) and mercaptoacetic acid, when refluxed in absolute ethanol. IR spectrum of compound (**12**) revealed absorption bands at 3370, 2355, 1730 and 1675 cm^–1^ of (NH), (SH) and two carbonyl groups, respectively. The ^13^ C–NMR spectra of compound (**12**) displayed signals at *δ* 20.3 and 30.1 ppm, agreeing to the two carbon atoms of CH_3_ and CH_2_ groups, and also at 166.4 and 181.2 ppm, matching the two carbon atoms of the two carbonyl groups. Moreover, Alkylation of thienotriazolopyrimidinone (**12**), in dry dimethylormamide and potassium hydroxide solution with ethylbromoacetate, afforded 2-benzoyl-3-methylthieno[2″,3″:4′,5′]pyrimido[1′,2′:2,3][1,2,4]triazolo[5,1-c][1,4]thiazine-4,7(8*H*,10*H*)-dione (**13**). ^13^C–NMR spectra of compound (**13**) showed signals at *δ* 20.5, 33.2 and 37.5 ppm, correspondent to three carbon atoms of one CH_3_ and two CH_2_ groups, respectively, and 165.9, 167.2 and 182.1 ppm, identical to three carbon atoms of the three carbonyl groups. In addition, the synthesis of 6-benzoyl-7-methyl-8-oxo-3, 8-dihydro- thieno[2,3-d][1,2,4]triazolo[1,5-a]pyrimidine-2-carboxylic acid (**14**) was synthesized in 72% yield from 2,3-diamino-benzoyl-methylthienopyrimidinone (**3**), oxalic acid and anhydrous potassium carbonate in dry dimethylformamide under refluxing conditions for 20–25 h. IR spectra of compound (**14**) showed absorption bands at 3375 and 3360 cm^–1^ of OH and NH groups and 1745, 1735 and 1679 cm^–1^ of three carbonyl groups. The ^1^H–NMR spectrum of compound (**14**) displayed two singlet broad signals at *δ* 10.20 and 12.45 ppm, corresponding to the two protons of the NH and OH groups (with D_2_O-exchangeable), respectively. Furthermore, condensation and cyclization of thienotriazolopyrimidinone (**12**), oxalic acid with the presence of anhydrous potassium carbonate in dry dimethyl- formamide gave 2-benzoyl-8-hydroxy-9-mercapto-3-methylpyrrolo[1′,2′:2,3][1,2,4] triazolo[1,5-a]thieno[2,3-d]pyrimidin-4,7-dione (**15**). The ^1^H–NMR spectrum of compound (**15**) showed two singlet signals at *δ* 2.70 and 12.10 ppm, agreeing to the two protons of the SH and OH groups (D_2_O-exchangeable). The mass spectrum of compounds **12**, **13**, **14** and **15** showed molecular ion peaks at m/z 356 (M^+^, 100%), 396 (M^+^, 95%), 354 (M^+^, 90%) and 410 (M^+^, 98%), respectively, as shown in Scheme 3. 

**Scheme 4:**
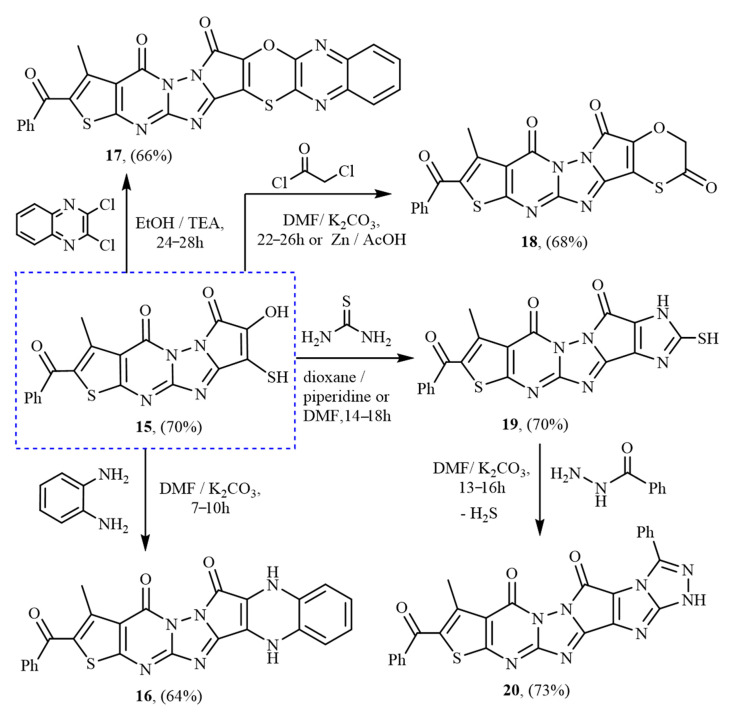
Synthesis of polycyclic: thienopyrimidotriazolopyrrolo; -quinoxaline, 1,4-oxathiino and imidazole derivatives.

Additionally, the treatment of pyrrolotriazolothienopyrimidine-4,7-dione (**15**) with *o*-phenylene-diamine in refluxing dimethylformamide and anhydrous potassium carbonate resulted in the formation of 2-benzoyl-3-methyl-8,13-dihydrothieno[2‴,3‴:4″,5″] pyrimido[1″,2″:2′,3′][1,2,4]triazolo [1′,5′:1,2]pyrrolo[3,4-b]quinoxaline-4,7-dione (**16**). The ^1^H NMR spectra of compound **16** exhibited two singlet broad signals at *δ* 11.50 and 11.57 ppm, conforming to the two protons of the two (2NH) groups (D_2_O-exchangeable). Likewise, 2-Benzoyl-3-Methylthieno[2⁗,3⁗:4‴,5‴]Pyrimido[1‴,2‴:2**″**,3**″**][1,2,4]Triazolo [1**″**, 5**″**:1′,2′]Pyrrolo[3′,4′:5,6][1,4]Oxathiino[2,3-b]Quinoxaline-4,7-Dione (**17**) was synthesized from the reaction of compound **15** with 2,3-dichloroquinoxaline in boiling absolute ethanol with the presence of a catalytic amount of triethylamine. In the ^13^C–NMR spectrum of compound **17**, it displayed signals at *δ* 166.5, 170.9 and 183.8 ppm, due to three carbonyl groups. The pyrrolotriazolothienopyrimidine-4,7-dione (**15**) also reacted with α-halo-ketones as chloroacetyl-chloride in dimethylformamide with the presence of anhydrous potassium carbonate or in acetic acid with the presence of zinc dust to afford the corresponding 8-benzoyl-9-methyl-[1,4]oxathiino[3″,2″:3′,4′]pyrrolo[1′,2′:2,3][1,2,4]triazo- lo[1,5-a]thieno[2,3-d]pyrimidine-3,10,13 (2*H*)-trione (**18**). The ^1^H–NMR spectrum of compound **18** showed one singlet signal at 5.10 ppm, conforming to the two protons of the CH_2_, and multiple singlet signals at *δ* 7.61–7.90 ppm, agreeing to the five protons of the phenyl group. Moreover, the reaction of pyrrolotriazolothienopyrimidine-4,7-dione (**15**) with thiourea in a mixture of dimethylformamide or dioxane in the presence of catalytic amount of piperidine under control (TLC) afforded the 7-benzoyl-2-mercapto-8- methyl-1*H*-imidazo[4″,5″:3′,4′]pyrrolo[1′,2′:2,3][1,2,4]triazolo[1,5-a]thieno[2,3-d]pyrimidi- ne-9, 12-dione (**19**). The IR spectrum of compound **19** displayed the presence of broad band absorption at *ν* 3355 cm^−1^ of one (NH) group, 2357 cm^−1^ of one (SH) group and 1729, 1681 and 1670 cm^−1^ of the three carbonyl groups. The ^1^H–NMR spectrum of compound **19** demonstrated two singlet broad signals at *δ* 10.83 and 11.90 ppm, corresponding to the two protons of the (SH) and (NH) groups, respectively (D_2_O-exchangeable). Furthermore, refluxed **19** with benzo-hydrazide in dry dimethylformamide and anhydrous potassium carbonate formed a new heterocyclic compound, 10- benzoyl-9-methyl-3-phenyl- 1*H*-[1,2,4]triazolo[4‴,3‴:1″,2″]imidazo[4″,5″:3′,4′]pyrrolo[1′,2′:2,3][1,2,4]triazolo[1,5-a]thie- no[2,3-d]pyrimidine-5,8-dione (**20**). The ^1^H–NMR spectrum of compound **20** revealed one singlet signal at *δ* 12.01 ppm for one proton of NH (D_2_O-exchangeable). The mass spectra of compounds **16**–**20** exhibited the molecular ion peaks at m/z 466 (M^+^, 100%), 536 (M^+^, 94%), 450 (M^+^, 90%), 434 (M^+^, 85%) and 518 (M^+^, 100%), respectively, as shown in Scheme 4.

### 2.2. Biological Activities

#### 2.2.1. Anticancer Screening (In Vitro Cytotoxicity)

The new compounds that were synthesized, such as thieno[2,3-d]pyrimidinones, thienotriazolopyrimidinones, thienopyrimido-triazolo-thiazines, pyrrolo-triazolothieno- pyrimidines, thieno-pyrimido-triazolopyrrolo-quinoxalines, thieno-pyrimidotriazolo-pyrrolo-oxathiino-quinoxalines, 1,4-oxathiino-pyrrolotriazolo-thienopyrimidin-triones, imidazopyrrolotriazolothienopyrimidin-diones and 1,2,4-triazoloimidazopyrrolotriazol- othienopyrimidin-diones, were tested for their in vitro cytotoxicity using the standard method to test cell growth rate and toxicity of the culture (MTT) method [34,38,40,48] against the human nasopharyngeal carcinoma cells (**CNE2**), oral carcinoma cells (**KB**), breast adenocarcinoma cells (**MCF-7**) and gastric carcinoma cells (**MGC-803**). The MTT method is based on the reduction of soluble 3-(4,5-dimethyl-2-thiazolyl)-2,5-diphenyl-2*H*-tetrazolium bromide, as shown in Table 1. Through the final results of the effect of compounds against the types of human carcinoma cell lines, we found many compounds, such as **20**–**19**, **17**–**16**, **11** and **18** gave the highest anticancer activity. Some of the compounds, **20**–**19**, exhibited the highest effect of cytotoxicity and outperformed the 5-Fluorouracil-positive control. Many compounds had moderate results in activity against cancer cells, such as **15**, **5**, **7**, **13** and **12** and the rest of the compounds in the manuscript, had a weak effect against cancer cells. Consequently, the compounds **20**, **19**, **17**, **16**, **11** and **18** possess the best activities against all carcinoma cell lines as follows: the **CNE2** (IC_50_: Comp. **20**–**19**, **17**–**16**, **11** and **18** gives 11.6–11.7, 11.8–11.9, 12.1 and 12.4 μM); the **KB** (IC_50_: Comp. **20**–**19**, **17**–**16**, **11** and **18** gives 10.5, 10.7, 10.8–10.9, 11.1 and 11.3 μM); the **MCF-7** (IC_50_: Comp. **20**–**19**, **17**–**16**, **11** and **18** gives 11.3–11.4, 11.5–11.6, 11.8 and 11.9 μM); the **MGC-803** (IC_50_: Comp. **20**–**19**, **17**–**16**, **11** and **18** gives 10.9, 11.1, 11.3, 11.5, 11.7 and 11.9 μM), respectively. All results of the compounds as anticancer activities are mentioned in Table 1.

#### 2.2.2. Structural Activity Relationship (SAR)

The relationship of the chemical structures (SARs) of the prepared compounds to the results mentioned in this study, in terms of the ability of these compounds to apply cytotoxicity activity to human carcinoma cells, is due to several possibilities, including the following: (**A**) The presence of the thienopyrimidinones, 1,2,4-triazolopyrimidinone, 1,4-thiazine, pyrrolo-1,2,4-triazole, pyrroloquinoxaline, oxathiinoquinoxaline, 1,4-oxa-thiinopyrrolotriazole, imidazopyrrolotriazole and 1,2,4-triazoloimidazopyrrolo-triazole moieties may be requested for a broad spectrum of the cytotoxicity activity. (**B**) 1,2,4-triazoloimidazopyrrolotriazolothienopyrimidindione (**20**), imidazopyrrolotriazolo- thienopyrimidindione (**19**), thienopyrimidotriazolopyrrolooxathiinoquinoxalindione (**17**), thieno-pyrimidotriazolopyrroloquinoxalindione (**16**), 2-(((6-benzoyl-thienotriazolo- pyrimidin-2-yl)methyl) thio)-thienotriazolopyrimidinene (**11**) and 1,4-oxathiinopyrrolo- triazolothienopyrimidin-trione (**18**) demonstrated great anticancer activity in vitro, and this corresponds to previous scientific studies, because numerous heterocyclic compounds have pharmacological activities, such as imidazoles, benzimidazoles, thieno-[2,3-d]pyrimidines, 1,2,4-triazoles, thiazolopyrimidines, pyrrolothiazolo-pyrimidines, pyrrolotriazines, pyrrolotriazepines, triazolopyrrolothiazolopyrimidines, quinolines, quinoxalines, thiazolidinones, thiazines, thienotriazoles, thiophenes, phenyl, benzoyl and methyl, which have displayed potent anticancer activity [2,5,8,10,20,25,34,38,40,48,49,50]. (**C**) Thence, the compounds **20**, **19**, **17**, **16**, **11** and **18** offered high cytotoxicity against all human carcinoma cell lines, such as nasopharyngeal (**CNE2**), oral (**KB**), breast (**MCF-7**) and gastric (**MGC-803**), during the comparison with 5-Fluorouracil as a standard drug, shown in Table 2. From studying the chemical structures of compounds **20**–**16** and **11**, it becomes clear that these compounds are preferred for the following reasons: (**I**) they contain thienotriazolopyrimidinene as the basis in these compounds. (**II**) Some compounds containing pyrrole, imidazole and triazole rings are all linked with the thienotriazolopyrimidinene basis, such as compound **20**. (**III**) Some compounds that contain pyrrole and imidazole rings are all linked with the thienotriazolopyrimidinene basis, such as compound **19**. (**IV**) Some compounds that contain pyrrole, oxathiine and quinoxaline rings are all linked with the thienotriazolopyrimidinene basis, such as compound **17**. (**V**) Some compounds that contain pyrrole and quinoxaline rings are all linked with the thienotriazolopyrimidinene basis, such as compound **16**. (**VI**) Some compounds that contain pyrrole and oxathiine rings are all linked with the thienotriazolopyrimidinene basis, such as compound **18**. (**VII**) Some compounds linked with two molecules of thienotriazolopyrimidinene, such as compound **11**. (**VIII**) These are in addition to many functional groups, such as methyl and phenyl, and many heteroatoms, such as nitrogen, oxygen and sulfur.

## 3. Experimental Section

### 3.1. General Information

All melting points were taken on an Electrothermal IA 9100 series digital melting point apparatus (Shimadzu, Tokyo, Japan). Elemental analyses were performed on Vario EL (Elementar, Langenselbold, Germany). Microanalytical data were processed in the microanalytical center, Faculty of Science, Cairo University and National Research Centre. The IR spectra (KBr disc) were recorded using a Perkin–Elmer 1650 spectrometer (Waltham, MA, USA). NMR spectra were determined using JEOL 270 MHz and JEOL JMS-AX 500 MHz (JEOL, Tokyo, Japan) spectrometers with Me4Si as an internal standard. Mass spectra were recorded on an EI Ms-QP 1000 EX instrument (Shimadzu, Tokyo, Japan) at 70 eV. Biological evaluations were done by the anticancer unit, Mansoura University, Faculty of Pharmacy (Department of Pharmacognosy), 35516, Egypt. All starting materials and solvents were purchased from Sigma–Aldrich (Saint Louis, MO, USA).

### 3.2. Synthesis of 2,3-Diamino-6-Benzoyl-5-Methylthieno[2,3-d]Pyrimidin-4(3H)-One *(**3**)*

Compound **2** (3.42 g, 0.01 mol) in 15 mL of hydrazine hydrate was heated under reflux for 7 h. The reaction mixture was cooled and suspended in 50 mL of water. The solid was collected by suction filtration, washed with water, and recrystallized from ethanol to afford compound **3** (75%). M. p.: 270–272 °C. IR (KBr): *ν*_max_ = 3400, 3385 (2NH_2_), 1650, 1622 (2CO), 1585 (C=N) cm^−1^; ^1^H–NMR (500 MHz, DMSO-*d*_6_): *δ =* 2.20 (s, 3H, CH_3_), 6.41 (br. s, 2H, CNH_2_), 7.43–7.94 (m, 5H, Ar-H), 11.49 (br. s, 2H, NNH_2_) ppm; ^13^C–NMR (125.7 MHz, DMSO-*d*_6_): *δ =* 19.2, 121.3, 128.6, 129.3, 131.5, 132.3, 135.3, 142.2, 146.4, 149.5, 158.1, 168.9 ppm; MS (70 eV): m/z = 300 (M^+^, 100%); Anal. Calc. (Found) for C_14_H_12_N_4_O_2_S (300.34): C, 55.99 (55.90); H, 4.03 (4.10); N, 18.66 (18.60).

### 3.3. Synthesis of 2-(4-Aminophenyl)-6-Benzoyl-7-Methylthieno[2,3-d][1,2,4]Triazolo[1,5-a]Pyrimidin-8(3H)-One *(**4**)*

Equimolar quantities of 2,3-diamino-benzoyl-methylthienopyrimidinone (**3**) (3 g, 0.01 mol) and 4-aminobenzoic acid (1.37 g, 0.01 mol) were dissolved in polyphosphoric acid (30 mL) and refluxed for 18–20 h at 250–280 °C under control (TLC), with constant stirring. The reaction mixtures were cooled and slowly poured into crushed ice with constant stirring. The pH of the reaction mixture was detected to alkaline by adding 4N of anhydrous sodium carbonate. The final precipitates were filtered under pressure, washed with distilled water and recrystallized from ethanol as yellow crystals in 70% yield, m. p. > 350 °C. IR (KBr): *ν*_max_ = 3405, 3355 (NH_2,_ NH), 3045 (CH, aryl), 2940 (CH, alkyl), 1680, 1665 (2C=O), 1620 (C=N), 1585 (C=C) cm^−1^; ^1^H–NMR (DMSO-*d*_6_): *δ* = 2.10 (s, 3H, CH_3_), 6.10 (br. s, 2H, NH_2_), 7.50–7.90 (m, 9H, Ar-H), 10.70 (br,1H, NH, D_2_O-exchangeable); ^13^C–NMR (DMSO-*d*_6_) *δ* = 20.4 (1C, CH_3_), 115.1, 119.4, 121.3, 127.1, 128.5, 129.9, 131.5, 135.7, 140.9, 143.1, 147.2, 148.5, 151.8, 162.9 (18C, Ar-C), 165.1, 181.4 (2C, 2C=O); MS (70 eV): m/z = 401 (M^+^, 100%); Anal. Calc. (Found) for C_21_H_15_N_5_O_2_S (401.44): C, 62.83 (62.75); H, 3.77 (3.70); N, 17.45 (17.64).

### 3.4. Synthesis of N-(4-(3-Acetyl-6-Benzoyl-7-Methyl-8-oxo-3,8-Dihydrothieno[2,3-d][1,2,4] Triazolo[1,5-a]Pyrimidin-2-yl)Phenyl) Acetamide *(**5**)*

**Method A:** To a warm (60–80 °C) solution of sodium ethoxide (prepared by dissolving 0.23 g of sodium metal in 35 mL ethanol), compound **4** (4.01 g, 10 mmol) was added, and heating (60–80 °C) was continued for 40 min. The mixture was allowed to cool to room temperature, and acetic anhydride (10 mmol) was added. The mixture was stirred under reflux for 8–10 h, allowed to cool to room temperature and finally poured into 100 mL cold water. The solid product precipitate was filtered off and washed with 100 mL water. The solid obtained was filtered off, dried and crystallized from the proper solvent to produce compound **5**. **Method B:** A mix of compound **4** (4.01 g, 0.01 mol) and acetic anhydride (30 mL) was refluxed with heat for one hour, checked via (TLC). After cooling, the solution was concentrated and poured into crushed ice. The separated solid was filtered off, dried and recrystallized from benzene as yellowish crystals in 74% yield, m. p. > 350 °C. IR (KBr): *ν*_max_ = 3325 (NH), 3040 (CH, aryl), 2935 (CH, alkyl), 1705, 1685, 1680, 1675 (4C=O), 1622 (C=N), 1580 (C=C) cm^−1^; ^1^H–NMR (DMSO-*d*_6_): *δ* = 1.90, 2.05, 2.15 (3s, 9H, 3CH_3_), 7.52–7.93 (m, 9H, Ar-H), 11.90 (br, s.1H, NH, D_2_O-exchangeable); ^13^C–NMR (DMSO-*d*_6_) *δ* = 20.7, 22.5, 26.3 (3C, 3CH_3_), 118.8, 122.1, 123.4, 126.7, 128.6, 129.5, 132.2, 136.8, 139.5, 141.4, 143.7, 148.5, 152.2, 163.5 (18C, Ar-C), 164.8, 169.2, 175.1, 182.7 (4C, 4C=O); MS (70 eV): m/z = 485 (M^+^, 100%); Anal. Calc. (Found) for C_25_H_19_N_5_O_4_S (485.52): C, 61.85 (61.77); H, 3.94 (3.85); N, 14.42 (14.51).

### 3.5. General Procedure for Synthesis of 6-Benzoyl-2-(4-Substituted-Phenyl)-7-Methylthieno[2,3-d][1,2,4]Triazolo[1,5-a]Pyrimidin-8(3H)-One *(**6a**–**c**)*

A mixture of 2,3-diamino-benzoyl-methylthienopyrimidinone (**3**) (3 g, 0.01 mol), the appropriate aromatic aldehyde (0.01 mol) and the catalytic amount of potassium iodide (0.3 g, 0.002 mol) in dimethyl- formamide (40 mL) was heated and refluxed for 20–22 h at 180–200 °C; the progress of reaction was monitored by TLC, and, at the end of reaction, the solvent was allowed to cool to room temperature and poured into water (100 mL). Then the obtained solid products were filtered off, dried and crystallized from the appropriate solvent to give compounds **6a**–**c** in good yields, respectively.

### 3.6. Synthesis of 6-Benzoyl-7-Methyl-2-Phenylthieno[2,3-d][1,2,4]Triazolo[1,5-a]Pyrimidin-8(3H)-One *(**6a**)*

The compound was obtained from the reaction of compound **3** and benzaldehyde (1.06 g, 0.01 mol) with DMF/KI, as white crystals, crystallized from dioxane in 70% yield, m. p. > 350 °C. IR (KBr): *ν*_max_ = 3350 (NH), 3060 (CH, aryl), 2937 (CH, alkyl), 1710, 1672 (2C=O), 1628 (C=N), 1580 (C=C) cm^−1^; ^1^H–NMR (DMSO-*d*_6_): *δ* = 2.17 (s, 3H, CH_3_), 7.40–7.84 (m, 10H, Ar-H), 10.55 (br,1H, NH, D_2_O-exchangeable); ^13^C–NMR (DMSO-*d*_6_) *δ* = 20.2 (1C, CH_3_), 121.2, 128.1, 128.6, 128.7, 128.9, 129.5, 130.4, 132.1, 138.7, 142.4, 143.6, 148.5, 151.9, 162.2 (18C, Ar-C), 164.9, 182.7 (2C, 2C=O); MS (70 eV): m/z = 386 (M^+^, 90%); Anal. Calc. (Found) for C_21_H_14_N_4_O_2_S (386.43): C, 65.27 (65.20); H, 3.65 (3.74); N, 14.50 (14.40).

### 3.7. Synthesis of 6-Benzoyl-2-(4-Chlorophenyl)-7-Methylthieno[2,3-d][1,2,4]Triazolo[1,5-a]Pyrimidin-8(3H)-One *(**6b**)*

The compound was obtained from the reaction of compound **3** and 4-chlorobenzaldehyde (1.40 g, 0.01 mol) with DMF/KI, as white crystals, crystallized from methanol in 80% yield, m. p. > 350 °C. IR (KBr): *ν*_max_ = 3355 (NH), 3065 (CH, aryl), 2940 (CH, alkyl), 1715, 1670 (2C=O), 1630 (C=N), 1582 (C=C) cm^−1^; ^1^H–NMR (DMSO-*d*_6_): *δ* = 2.20 (s, 3H, CH_3_), 7.35–7.41 (dd, 2H, J = 7.50, 7.53 Hz, 4-chlorophenyl), 7.46–7.52 (dd, 2H, J = 7.54, 7.58 Hz, 4-chlorophenyl), 7.62–7.90 (m, 5H, Ar-H), 10.66 (br,1H, NH, D_2_O-exchangeable); ^13^C–NMR (DMSO-*d*_6_) *δ* = 20.5 (1C, CH_3_), 121.7, 126.8, 128.5, 128.7, 129.4, 129.7, 132.3, 135.6, 137.1, 142.3, 143.2, 148.7, 152.3, 163.1 (18C, Ar-C), 165.5, 183.1 (2C, 2C=O); MS (70 eV): m/z = 420 (M^+^, 100%); Anal. Calc. (Found) for C_21_H_13_ClN_4_O_2_S (420.87): C, 59.93 (59.85); H, 3.11 (3.19); N, 13.31 (13.39).

### 3.8. Synthesis of 6-Benzoyl-2-(4-Methoxyphenyl)-7-Methylthieno[2,3-d][1,2,4]Triazolo[1,5-a]Pyrimidin-8(3H)-One *(**6c**)*

The compound was obtained from the reaction of compound **3** and 4-methoxy- benzaldehyde (1.36 g, 0.01 mol) with DMF/KI, as yellowish crystals, crystallized from benzene in 72% yield, m. p. > 350 °C. IR (KBr): *ν*_max_ = 3358 (NH), 3067 (CH, aryl), 2945 (CH, alkyl), 1718, 1674 (2C=O), 1633 (C=N), 1585 (C=C) cm^−1^; ^1^H–NMR (DMSO-*d*_6_): *δ* = 2.25 (s, 3H, CH_3_), 3.75 (s, 3H, OCH_3_), 7.05–7.12 (dd, 2H, J = 7.51, 7.54 Hz, 4- methoxyphenyl), 7.32–7.39 (dd, 2H, J = 7.56, 7.59 Hz, 4- methoxyphenyl), 7.65–7.95 (m, 5H, Ar-H), 10.72 (br,1H, NH, D_2_O-exchangeable); ^13^C–NMR (DMSO-*d*_6_) *δ* = 21.1, 58.4 (2C, 2CH_3_), 121.9, 122.2, 124.5, 128.4, 128.9, 129.8, 132.5, 136.7, 142.3, 143.6, 148.2, 152.5, 161.3, 163.4, (18C, Ar-C), 166.1, 183.8 (2C, 2C=O); MS (70 eV): m/z = 416 (M^+^, 95%); Anal. Calc. (Found) for C_22_H_16_N_4_O_3_S (416.45): C, 63.45 (63.52); H, 3.87 (3.79); N, 13.45 (13.37).

### 3.9. Synthesis of 6-Benzoyl-7-Methyl-2-(Phenylamino)Thieno[2,3-d][1,2,4]Triazolo[1,5-a]Pyrimidin-8(3H)-One *(**7**)*

To a suspension of 2,3-diamino-benzoyl-methylthienopyrimidinone (**3**) (3 g, 0.01 mol) and red mercury (II) oxide (2.16 g, 0.01 mol) in 40 mL of dimethylformamide was added 1.83 g, 0.01 mol, of the corresponding methylphenylcarbamodithioate in 5 mL of DMF at room temperature. The mixture was refluxed for 15–18 h under control (TLC). After cooling, the mixture was filtrated, and to the filtrate were added 50 mL of water. The precipitate thus obtained was filtrated off, washed with water, dried and recrystallized from dioxane in 76% yield as yellow crystals, m. p. > 350 °C. IR (KBr): *ν*_max_ = 3360, 3320 (2NH), 3065 (CH, aryl), 2940 (CH, alkyl), 1715, 1676 (2C=O), 1630(C=N), 1585 (C=C) cm^−1^; ^1^H–NMR (DMSO-*d*_6_): *δ* = 2.20 (s, 3H, CH_3_), 7.15–7.80 (m, 10H, Ar-H), 9.60, 10.80 (br, 2H, 2NH, D_2_O-exchangeable); ^13^C–NMR (DMSO-*d*_6_) *δ* = 20.7 (1C, CH_3_), 120.8, 122.1, 123.4, 128.7, 129.2, 129.8, 131.6, 137.5, 137.9, 142.4, 143.1, 143.9, 152.3, 161.9 (18C, Ar-C), 163.8, 181.4 (2C, 2C=O); MS (70 eV): m/z = 401 (M^+^, 95%); Anal. Calc. (Found) for C_21_H_15_N_5_O_2_S (401.44): C, 62.83 (62.75); H, 3.77 (3.70); N, 17.45 (17.52).

### 3.10. Synthesis of 6-Benzoyl-7-Methylthieno[2,3-d][1,2,4]Triazolo[1,5-a]Pyrimidine-2,8(1H,3H)-Dione *(**8**)*

To a solution of 2,3-diamino-benzoyl-methylthienopyrimidinone (**3**) (3 g, 0.01 mol) and anhydrous potassium carbonate (1.4 g, 0.01 mol) in dry acetone (50 mL), ethyl- chloroformate (1 mL, 0.01 mol) was added to the solution dropwise; after the completion of the addition, the mixture was refluxed for 14–17 h, allowed to cool at room temperature, and then poured into the cool water. The final product was obtained by being filtrated off, washed with ethanol and recrystallized from methanol in 84% yield as yellowish crystals, m. p. > 350 °C. IR (KBr): *ν*_max_ =3352, 3310 (2NH), 3050 (CH, aryl), 2945 (CH, alkyl), 1722, 1685, 1674 (3C=O), 1632 (C=N), 1588 (C=C) cm^−1^; ^1^H–NMR (DMSO-*d*_6_): *δ* = 2.23 (s, 3H, CH_3_), 7.55–7.77 (m, 5H, Ar-H), 10.20, 11.10 (br, 2H, 2NH, D_2_O-exchangeable); ^13^C–NMR (DMSO-*d*_6_) *δ* = 21.1 (1C, CH_3_), 121.1, 128.4, 129.2, 131.8, 138.1, 142.6, 143.7, 152.2, 162.5 (11C, Ar-C), 163.5, 166.2, 182.7 (3C, 3C=O); MS (70 eV): m/z = 326 (M^+^, 100%); Anal. Calc. (Found) for C_15_H_10_N_4_O_3_S (326.33): C, 55.21 (55.15); H, 3.09 (3.17); N, 17.17 (17.26).

### 3.11. Synthesis of 6-Benzoyl-2-Mercapto-7-Methylthieno[2,3-d][1,2,4]Triazolo[1,5-a]Pyrimidin-8(3H)-One *(**9**)*

KOH (0.56g, 0.01mol) was dissolved through stirring in 20 mL of anhydrous methanol in a 250 mL flask. Carbon disulfide (0.89 g, 0.01 mol) was dissolved in anhydrous methanol (5 mL) and added dropwise to the stirring solution, followed by reflux. The solution of 2,3-diamino-benzoyl-methyl- thienopyrimidinone (**3**) (3 g, 0.01 mol) in methanol (30 mL) was added to the above reaction mixture and stirring under reflux (75–80 °C) for 8–10 h. Initially, the solution was yellow, which then slowly turned to brown, and, as the reaction progressed, the evolution of hydrogen sulfide was observed. The reaction mixture was monitored via TLC (Ethyl-acetate: petroleum ether, 1:3). After completion of the reaction, the mixture was poured into a beaker containing ice water and acidified with 4N, HCl to maintain pH 4–5. The final precipitate was separated, then filtered and recrystallized from the benzene in 70% yield as yellow crystals, m. p. > 350 °C. IR (KBr): *ν*_max_ = 3345 (NH), 3040 (CH, aryl), 2937 (CH, alkyl), 2372 (SH), 1718, 1678 (2C=O), 1627(C=N), 1584(C=C) cm^−1^; ^1^H–NMR (DMSO-*d*_6_): *δ* = 2.17 (s, 3H, CH_3_), 2.62 (s, 1H, SH), 7.61–7.88 (m, 5H, Ar-H), 9.05 (br, 1H, NH, D_2_O-exchangeable); ^13^C–NMR (DMSO-*d*_6_) *δ* = 19.8 (1C, CH_3_), 120.7, 128.7, 129.5, 132.1, 138.6, 142.3, 143.5, 148.9, 151.8, 161.7 (12C, Ar-C), 164.5, 181.4 (2C, 2C=O); MS (70 eV): m/z = 342 (M^+^, 93%); Anal. Calc. (Found) for C_15_H_10_N_4_O_2_S_2_ (342.39): C, 52.62 (52.55); H, 2.94 (2.86); N, 16.36 (16.45).

### 3.12. Synthesis of 6-Benzoyl-2-(Chloromethyl)-7-Methylthieno[2,3-d][1,2,4]Triazolo[1,5-a]Pyrimidin-8(3H)-One *(**10**)*

Chloroacetic acid (0.95 g, 0.01 mol) was dissolved in 35 mL of 4N, HCl and stirred into the mixture for nearly 30 min. 2,3-diamino-benzoyl-methylthienopyrimidinone (**3**) (3 g, 0.01 mol) was added to the above solution with constant stirring; we continued stirring with reflux (100 °C) for nearly 7–9 h. The solid product was established by TLC (Ethyl acetate: petroleum ether, 1:1). The above hot solution was poured into ice cold water with stirring, followed by dropwise addition of con NH_3_; then the yellowish precipitate was filtered. The precipitate was then recrystallized from the dioxane in 79% yield as yellowish crystals, m. p. > 350 °C. IR (KBr): *ν*_max_ = 3341 (NH), 3035 (CH, aryl), 2933 (CH, alkyl), 1724, 1680 (2C=O), 1625 (C=N), 1581(C=C) cm^−1^; ^1^H–NMR (DMSO-*d*_6_): *δ* = 2.23 (s, 3H, CH_3_), 4.45 (s, 2H, CH_2_), 7.60–7.86 (m, 5H, Ar-H), 9.15 (br, 1H, NH, D_2_O-exchangeable); ^13^C–NMR (DMSO-*d*_6_) *δ* = 20.5 (1C, CH_3_), 50.2 (1C, CH_2_), 120.4, 128.4, 129.2, 132.3, 138.1, 142.5, 143.7, 152.2, 155.9, 162.5 (12C, Ar-C), 165.3, 181.8 (2C, 2C=O); MS (70 eV): m/z = 358 (M^+^, 91%); Anal. Calc. (Found) for C_16_H_11_ClN_4_O_2_S (358.80): C, 53.56 (53.50); H, 3.09 (3.15); N, 15.62 (15.71).

### 3.13. Synthesis of 6-Benzoyl-2-(((6-Benzoyl-7-Methyl-8-oxo-3,8-Dihydrothieno[2,3-d][1,2,4]Triazolo[1,5-a]Pyrimidin-2-yl)Methyl)thio)-7-Methylthieno[2,3-d][1,2,4]Triazolo[1,5-a]Pyrimidin-8(3H)-One *(**11**)*

The 0.22 g, 0.01 mol, of sodium metal was added to a solution of 2-mercapto-methyl- thieno-1,2,4-triazolopyrimidine (**9**) (3.42 g, 0.01 mol) in 40 mL anhydrous methanol and stirred vigorously for 35 min. The 2-chloromethyl-methylthieno- 1,2,4-triazolopyrimi- dine (**10**) (3.58g, 0.01 mol) was added to the above reaction mixture in portions and continually stirred for (90–100 °C) nearly 5–8 h. A yellow compound so obtained was filtered, washed with the methanol and dried. The product was established via TLC (Ethyl acetate: petroleum ether, 1:1). The solid precipitate was then filtered and recrystallized from the DMF in 68% yield as yellow crystals, m. p. > 350 °C. IR (KBr): *ν*_max_ = 3355 (2NH), 3050 (CH, aryl), 2940 (CH, alkyl), 1730, 1728, 1683, 1675 (4C=O), 1628 (C=N), 1585 (C=C) cm^−1^; ^1^H–NMR (DMSO-*d*_6_): *δ* = 2.16, 2.28 (s, 6H, 2CH_3_), 4.05 (s, 2H, CH_2_), 7.35–7.95 (m, 10H, Ar-H), 9.80, 10.10 (br, 2H, 2NH, D_2_O-exchangeable); ^13^C–NMR (DMSO-*d*_6_) *δ* = 20.3, 20.8 (2C, 2CH_3_), 33.7 (1C, CH_2_), 120.6, 120.9, 128.2, 128.5, 129.4, 129.8, 132.5, 132.7, 138.3, 138.6, 142.1, 142.5, 143.2, 143.6, 144.3, 152.4, 152.8, 156.1, 163.1, 163.4 (24C, Ar-C), 166.1, 166.5, 182.2, 182.8 (4C, 4C=O); MS (70 eV): m/z = 664 (M^+^, 90%); Anal. Calc. (Found) for C_31_H_20_N_8_O_4_S_3_ (664.73): C, 56.01 (56.12); H, 3.03 (3.10); N, 16.86 (16.73).

### 3.14. Synthesis of 6-Benzoyl-2-(Mercaptomethyl)-7-Methylthieno[2,3-d][1,2,4]Triazolo[1,5-a]Pyrimidin-8(1H)-One *(**12**)*

A mixture of 2,3-diamino-benzoyl-methylthienopyrimidinone (**3**) (3 g, 0.01 mol) and thioglycolic acid (0.92 g, 0.01 mol) in absolute ethanol (25 mL). The reaction solution was heated under reflux for 8–10 h by under control (TLC), then left to cool to room temperature. The separated crystalline product was filtered, washed with ethanol and crystallized from methanol in 73% yield as yellow crystals, m. p. > 350 °C. IR (KBr): *ν*_max_ = 3370 (NH), 3040 (CH, aryl), 2950 (CH, alkyl), 2355 (SH), 1730, 1675 (2C=O), 1629 (C=N), 1580 (C=C) cm^−1^; ^1^H–NMR (DMSO-*d*_6_): *δ* = 2.25 (s, 3H, CH_3_), 2.60 (s, 1H, SH), 4.25 (s, 2H, CH_2_), 7.55–7.82 (m, 5H, Ar-H), 10.10 (br, 1H, NH, D_2_O-exchangeable); ^13^C–NMR (DMSO-*d*_6_) *δ* = 20.3 (1C, CH_3_), 30.1 (1C, CH_2_), 120.1, 128.6, 129.5, 131.8, 137.6, 142.4, 143.5, 157.1, 158.8, 160.2 (12C, Ar-C), 166.4, 181.2 (2C, 2C=O); MS (70 eV): m/z = 356 (M^+^, 100%); Anal. Calc. (Found) for C_16_H_12_N_4_O_2_S_2_ (356.42): C, 53.92 (53.84); H, 3.39 (3.44); N, 15.72 (15.78).

### 3.15. Synthesis of 2-Benzoyl-3-Methylthieno[2″,3″:4′,5′]Pyrimido[1′,2′:2,3][1,2,4]Triazolo [5,1-c][1,4] Thiazine-4,7(8H,10H)-Dione *(**13**)*

A well-stirred and ice-cooled suspension of finely powdered potassium hydroxide (0.56 g, 0.01 mol) and thienotriazolopyrimidinone (**12**) (3.56 g, 0.01 mol) in dry dimethylformamide (30 mL). After complete addition, heating and stirring was continued at room temperature for 2–4 h. The reaction mixture was cooled to 0 °C, treated with the appropriate halogenated compound as ethylbromoacetate (1.10 mL, 0.01 mol), heated and stirred at room temperature for 18–22 h under control (TLC), then poured onto ice water. The resulting product was filtered off, dried and recrystallized from dimethylformamide in 80% yield as yellowish crystals, m. p. > 350 °C. IR (KBr): *ν*_max_ = 3050 (CH, aryl), 2955 (CH, alkyl), 1733, 1682, 1677 (3C=O), 1631 (C=N), 1582 (C=C) cm^−1^; ^1^H–NMR (DMSO-*d*_6_): *δ* = 2.28 (s, 3H, CH_3_), 3.25 (s, 2H, CH_2_, thiazine), 3.70 (s, 2H, CH_2_, thiazine), 7.60–7.88 (m, 5H, Ar-H); ^13^C–NMR (DMSO-*d*_6_) *δ* = 20.5 (1C, CH_3_), 33.2, 37.5 (2C, 2CH_2_), 120.4, 128.7, 129.6, 131.9, 137.8, 142.7, 143.6, 158.5, 159.6, 161.3 (12C, Ar-C), 165.9, 167.2, 182.1 (3C, 3C=O); MS (70 eV): m/z = 396 (M^+^, 95%); Anal. Calc. (Found) for C_18_H_12_N_4_O_3_S_2_ (396.44): C, 54.53 (54.62); H, 3.05 (3.13); N, 14.13 (14.05).

### 3.16. Synthesis of 6-Benzoyl-7-Methyl-8-oxo-3,8-Dihydrothieno[2,3-d][1,2,4]Triazolo[1,5-a]Pyrimidine-2-Carbox-ylic Acid *(**14**)*

**Method A:** A mixture of 2,3-diamino-benzoyl-methylthienopyrimidinone (**3**) (3 g, 0.01 mol), oxalic acid (0.9 g, 0.01 mol) and anhydrous potassium carbonate (1.4 g, 0.01 mol) in dry dimethyl- formamide (40 mL); the mixture was refluxed for 20–25 h under control (TLC). The final product was filtered off, washed with cold water, dried and crystallized from the proper solvent. **Method B**: A mixture of 2,3-diamino-benzoyl- methylthienopyrimidinone (**3**) (3 g, 0.01 mol) and oxalic acid (0.9 g, 0.01 mol) in a solution of phosphorus pentoxide and methanesulfonic acid (PPMA) at 110–125 °C for 22–27 h under control (TLC). (PPMA was prepared by adding phosphorus pentoxide (30 g) to methane sulfonic acid (210 mL) in portions and stirring the mixture at 80 °C until the phosphorus pentoxide had completely dissolved.) Then, the mixture was allowed to cool at room temperature and then poured into the cool water. The solid product was obtained by being filtrated off, washed with ethanol and recrystallized from dioxane in 72% yield as yellow crystals, m. p. > 350 °C. IR (KBr): *ν*_max_ = 3375 (br, OH), 3360 (NH), 3058 (CH, aryl), 2960 (CH, alkyl), 1745, 1735, 1679 (3C=O), 1630 (C=N), 1585 (C=C) cm^−1^; ^1^H–NMR (DMSO-*d*_6_): *δ* = 2.30 (s, 3H, CH_3_), 7.63–7.90 (m, 5H, Ar-H), 10.20 (br, 1H, NH, D_2_O-exchangeable), 12.45 (br, 1H, OH, D_2_O-exchangeable); ^13^C–NMR (DMSO-*d*_6_) *δ* = 20.8 (1C, CH_3_), 120.6, 128.9, 129.5, 132.1, 137.9, 142.4, 142.8, 143.8, 151.7, 160.9 (12C, Ar-C), 164.8, 169.1, 183.5 (3C, 3C=O); MS (70 eV): *m*/*z* = 354 (M^+^, 90%); Anal. Calc. (Found) for C_16_H_10_N_4_O_4_S (354.34): C, 54.23 (54.31); H, 2.84 (2.75); N, 15.81 (15.90).

### 3.17. Synthesis of 2-Benzoyl-8-Hydroxy-9-Mercapto-3-Methylpyrrolo[1′,2′:2,3][1,2,4]Triazolo[1,5-a]Thieno[2,3-d]Pyrimidine-4,7-Dione *(**15**)*

A solution of thienotriazolopyrimidinone (**12**) (3.56 g, 0.01 mol), oxalic acid (0.9 g, 0.01 mol) and anhydrous potassium carbonate (1.4 g, 0.01 mol) in dry dimethylformamide (45 mL). The reaction mixture was refluxed for 17–20 h under control (TLC). The formed precipitate was filtered off, washed with cold water, dried and crystallized from benzene in 70% yield as yellowish crystals, m. p. > 350 °C. IR (KBr): *ν*_max_ = 3380 (br, OH), 3070 (CH, aryl), 2955 (CH, alkyl), 2360 (SH), 1728, 1680, 1673 (3C=O), 1633 (C=N), 1591 (C=C) cm^−1^; ^1^H–NMR (DMSO-*d*_6_): *δ* = 2.32 (s, 3H, CH_3_), 2.70 (s, 1H, SH), 7.56–7.87 (m, 5H, Ar-H), 12.10 (br, 1H, OH, D_2_O-exchangeable); ^13^C–NMR (DMSO-*d*_6_) *δ* = 21.1 (1C, CH_3_), 98.5 (1C, C-SH, pyrrole), 120.4, 128.5, 129.4, 132.3, 138.6, 142.6, 143.5, 152.2, 159.7, 160.4 (12C, Ar-C), 178.7 (1C, C-OH, pyrrole) 165.1, 169.5, 183.9 (3C, 3C=O); MS (70 eV): m/z = 410 (M^+^, 98%); Anal. Calc. (Found) for C_18_H_10_N_4_O_4_S_2_ (410.42): C, 52.68 (52.75); H, 2.46 (2.40); N, 13.65 (13.71).

### 3.18. Synthesis of 2-Benzoyl-3-Methyl-8,13-Dihydrothieno[2‴,3‴:4″,5″]Pyrimido[1″,2″:2′,3′][1,2,4]triazolo[1′,5′:1,2]Pyrrolo[3,4-b]Quinoxaline-4,7-Dione *(**16**)*

A mixture of compound **15** (4.10 g, 0.01 mol) and anhydrous potassium carbonate (1.4 g, 0.01 mol) in dry dimethylformamide (40 mL) was refluxed for 7–10 h under control (TLC). The product formed was filtered off, washed with methanol, dried and recrystallized from dioxane in 64% yield as yellow crystals, m. p. > 350 °C. IR (KBr): *ν*_max_ = 3385–3370 (2NH), 3075 (CH, aryl), 2960 (CH, alkyl), 1735, 1685, 1675 (3C=O), 1635 (C=N), 1595 (C=C) cm^−1^; ^1^H–NMR (DMSO-*d*_6_): *δ* = 2.35 (s, 3H, CH_3_), 7.01–7.92 (m, 9H, Ar-H), 11.50, 11.57 (2s, 2H, 2NH, D_2_O-exchangeable); ^13^C–NMR (DMSO-*d*_6_) *δ* = 21.7 (1C, CH_3_), 114.2, 115.5, 119.1, 119.6, 120.4, 120.8, 128.9, 129.8, 132.5, 138.1, 142.5, 143.2, 152.8, 160.1, 162.4 (20 C, Ar-C), 166.7, 170.1, 183.5 (3C, 3C=O); MS (70 eV): m/z = 466 (M^+^, 100%); Anal. Calc. (Found) for C_24_H_14_N_6_O_3_S (466.48): C, 61.80 (61.73); H, 3.03 (3.10); N, 18.02 (18.08).

### 3.19. Synthesis of 2-Benzoyl-3-Methylthieno[2⁗,3⁗:4‴,5‴]Pyrimido[1‴,2‴:2″,3″][1,2,4]Triazolo[1″,5″:1′,2′]Pyrrolo[3′,4′:5,6][1,4]Oxathiino[2,3-b]Quinoxaline-4,7-Dione *(**17**)*

A mixture of compound **15** (4.10 g, 0.01 mol) and 2,3- dichloroquinoxaline (1.99 g, 0.01 mol) in absolute ethanol (40 mL) containing 0.3 mL of TEA as a catalyst was refluxed for 24–28 h under control (TLC). The reaction solution was cooled, and the deposited solid was filtered off, dried and recrystallized from DMF in 66% yield as yellow crystals, m. p. > 350 °C. IR (KBr): *ν*_max_ = 3080 (CH, aryl), 2960 (CH, alkyl), 1730, 1680, 1675 (3C=O), 1635 (C=N), 1595 (C=C) cm^−1^; ^1^H–NMR (DMSO-*d*_6_): *δ* = 2.37 (s, 3H, CH_3_), 7.25–7.92 (m, 9H, Ar-H); ^13^C–NMR (DMSO-*d*_6_) *δ* = 21.9 (1C, CH_3_), 119.5, 120.8, 127.1, 127.4, 128.5, 129.2, 129.8, 130.6, 132.4, 135.7, 137.9, 139.3, 140.5, 142.7, 143.5, 143.9, 149.6, 152.4, 160.8, 163.5 (22C, Ar-C), 166.5, 170.9, 183.8 (3C, 3C=O); MS (70 eV): m/z = 536 (M^+^, 94%); Anal. Calc. (Found) for C_26_H_12_N_6_O_4_S_2_ (536.54): C, 58.20 (58.28); H, 2.25 (2.16); N, 15.66 (15.74).

### 3.20. Synthesis of 8-Benzoyl-9-Methyl-[1,4]Oxathiino[3″,2″:3′,4′]Pyrrolo[1′,2′:2,3][1,2,4]Triazolo[1,5-a]Thieno[2,3-d]Pyrimidine-3,10,13(2H)-Trione *(**18**)*

**Method A:** A mix of compound **15** (4.10 g, 0.01 mol) and chloro-acetyl-chloride (1.13 g, 0.01 mol) was stirred under reflux in dimethylformamide (45 mL) in the presence of anhydrous potassium carbonate (0.015 mol) for 22–26 h under control (TLC). The reaction solution was allowed to cool to room temperature, poured into cooled water (100 mL) and neutralized. The final solid formed was filtered off, dried and crystallized from the appropriate solvent. **Method B:** To the stirring solution of compound **15** (4.10 g, 0.01 mol) and chloro-acetyl-chloride (1.13g, 0.01 mol) in glacial acetic acid (40 mL), activated zinc dust (1.3g, 0.02 mol) was added portion-wise at room temperature over a period of one hour. Stirring was continued for an additional 4–6 h. The reaction solution was heated on a water bath (90–95 °C) for 12–17 h under control (TLC). After allowing the reaction mixture to cool to room temperature, it was poured into cold water (100 mL). The solid precipitate was filtered, washed with water, dried and crystallized from DMF in 68% yield as yellowish crystals, m. p. > 350 °C. IR (KBr): *ν*_max_ = 3077 (CH, aryl), 2958 (CH, alkyl), 1737, 1727, 1679, 1672 (4C=O), 1631 (C=N), 1593 (C=C) cm^−1^; ^1^H–NMR (DMSO-*d*_6_): *δ* = 2.34 (s, 3H, CH_3_), 5.10 (s, 2H, CH_2_), 7.61–7.90 (m, 5H, Ar-H); ^13^C–NMR (DMSO-*d*_6_) *δ* = 21.3 (1C, CH_3_), 80.6 (1C, CH_2_, oxathiine), 90.2 (1C, CH, oxathiine), 120.4, 128.6, 129.5, 132.1, 138.5, 142.4, 143.1, 152.3, 155.8, 161.2, 162.7 (13C, Ar-C), 165.9, 171.6, 183.2, 192.5 (4C, 4C=O); MS (70 eV): m/z = 450 (M^+^, 90%); Anal. Calc. (Found) for C_20_H_10_N_4_O_5_S_2_ (450.44): C, 53.33 (53.42); H, 2.24 (2.17); N, 12.44 (12.36).

### 3.21. Synthesis of 7-Benzoyl-2-Mercapto-8-Methyl-1H-Imidazo[4″,5″:3′,4′]Pyrrolo[1′,2′:2,3][1,2,4]Triazolo[1,5-a]Thieno[2,3-d]Pyrimidine-9,12-Dione *(**19**)*

**Method A:** A mixture of compound **15** (4.10 g, 0.01 mol) and thiourea (0.76 g, 0.01 mol) in 45 mL of DMF was refluxed for 14–18 h under control (TLC). The final product formed was filtered off, washed with methanol, dried and recrystallized from a suitable solvent. **Method B:** A mix of compound **15** (4.10 g, 0.01 mol) and thiourea (0.76 g, 0.01 mol) was stirred under reflux in dioxane (45 mL) in the existence of the catalytic amount of piperidine (1 mL) for 18–22 h. The reaction solution was allowed to cooling at room temperature and poured into cooled water, and the final precipitate was filtered off, washed with methanol, dried and crystallized from DMF in 70% yield as brownish crystals, m. p. > 350 °C. IR (KBr): *ν*_max_ = 3355 (NH), 3050 (CH, aryl), 2945 (CH, alkyl), 2357(SH), 1729, 1681, 1670 (3C=O), 1626 (C=N), 1586 (C=C) cm^−1^; ^1^H–NMR (DMSO-*d*_6_): *δ* = 2.28 (s, 3H, CH_3_), 7.55–7.82 (m, 5H, Ar-H), 10.83 (s, 1H, SH), 11.90 (s, 1H, NH, D_2_O-exchangeable); ^13^C–NMR (DMSO-*d*_6_) *δ* = 21.1 (1C, CH_3_), 120.3, 128.4, 129.3, 130.1, 132.5, 137.8, 140.3, 142.7, 143.2, 157.4, 161.6, 163.2 (14C, Ar-C), 164.7 (1C,1C=O), 168.9 (1C, C-SH, imidazole), 170.2, 183.4 (2C, 2C=O); MS (70 eV): m/z = 434 (M^+^, 85%); Anal. Calc. (Found) for C_19_H_10_N_6_O_3_S_2_ (434.45): C, 52.53 (52.62); H, 2.32 (2.40); N, 19.34 (19.27).

### 3.22. Synthesis of 10-Benzoyl-9-Methyl-3-Phenyl-1H-[1,2,4]Triazolo[4‴,3‴:1″,2″]Imidazo[4″,5″:3′,4′]Pyrrolo[1′,2′:2,3][1,2,4]Triazolo[1,5-a]Thieno[2,3-d]Pyrimidine-5,8-Dione *(**20**)*

**Method A:** A mixture of compound **19** (4.34 g, 0.01 mol) and benzohydrazide (1.36 g, 0.01 mol) and anhydrous potassium carbonate (1.4 g, 0.01 mol) in dry dimethylformamide (40 mL). The reaction solution was heated under reflux for 13–16 h under control (TLC), and then allowed to cool and poured into ice water. The formed solid product was collected by filtration and crystallized from the proper solvent. **Method B:** A solution of compound **19** (4.34 g, 0.01 mol) and benzohydrazide (1.36 g, 0.01 mol) in ethanol (40 mL) containing sodium ethoxide [prepared by dissolving sodium metal (0.23 g, 0.01 mol) in ethanol] was heated under reflux for 11–15 h, the reaction mixture was cooled and the deposited precipitate was filtered off, washed with ice water/ethanol and acidified with 10% HCl. The formed precipitate was filtered, dried and crystallized from dioxane in 73% yield as yellowish crystals, m. p. > 350 °C. IR (KBr): *ν*_max_ = 3372 (NH), 3058 (CH, aryl), 2951 (CH, alkyl), 1733, 1684, 1676 (3C=O), 1631 (C=N), 1579 (C=C) cm^−1^; ^1^H–NMR (DMSO-*d*_6_): *δ* = 2.32 (s, 3H, CH_3_), 7.15–7.98 (m, 10H, Ar-H), 12.01 (s, 1H, NH, D_2_O-exchangeable); ^13^C–NMR (DMSO-*d*_6_) *δ* = 21.6 (1C, CH_3_), 119.2, 120.7, 127.8, 128.2, 129.4, 129.8, 130.5, 131.8, 132.9, 136.5, 138.7, 142.5, 143.7, 150.8, 151.2, 155.4, 160.9, 162.7 (22C, Ar-C), 165.1, 168.8, 183.4 (3C, 3C=O); MS (70 eV): m/z = 518 (M^+^, 100%); Anal. Calc. (Found) for C_26_H_14_N_8_O_3_S (518.51): C, 60.23 (60.33); H, 2.72 (2.65); N, 21.61 (21.68).

### 3.23. Pharmacological Screening

#### 3.23.1. Ethics Approval and Consent to Participate

No humans or animals were used in this study, nevertheless: all the procedures are carried out under the Medical Research Ethics Committee of Mansoura University, Faculty of Pharmacy, Department of Pharmacognosy, 35516, Egypt.

#### 3.23.2. Human and Animal Rights

No humans or animals were used in the study. The research was conducted according to ethical standards in vitro.

#### 3.23.3. Chemicals and Drugs

Types of human carcinoma cancer cell line (**CNE2**, **KB**, **MCF-7** and **MGC-803**) are derived from the National Cancer institute, Cairo University, Cairo, Egypt, and 5-Fluorouracil and DMSO were purchased from Sigma–Aldrich (Saint Louis, MO, USA).

#### 3.23.4. Materials and Methods (In Vitro Cytotoxicity)

The in vitro cytotoxicity of the synthesized compounds against different cancer cell lines was performed with the MTT assay, according to the method found in [34,38,40,48]. The MTT assay is based on the reduction of the soluble 3-(4,5-dimethyl-2-thiazolyl)-2,5-diphenyl-2*H*-tetrazoliumbromide (MTT) into a blue–purple formazan product, mainly by mitochondrial reductase activity inside living cells. The cells used in the cytotoxicity assay were cultured in the suitable cell culture medium (RPMI 1640) medium supplemented with 10% fetal calf serum. Cells suspended in the medium (2Y’ 104/mL) were plated in 96-well culture plates and incubated at 37 °C in a 5% CO_2_ incubator. After 12 h, the test sample (2 mL) was added to the cells (2Y’ 104) in 96-well plates and cultured at 37 °C for 3 days. The cultured cells were mixed with 20 mL of MTT solution and incubated for 4 h at 37 °C. The supernatant was carefully removed from each well, and 100 mL of DMSO was added to each well to dissolve the formazan crystals, which were formed by the cellular reduction of MTT. After mixing with a mechanical plate mixer, the absorbance of each well was measured by a microplate reader using a test wavelength of 570 nm. The results were expressed as the IC_50_, which is the concentration of the drugs inducing a 50% inhibition of cell growth of treated cells, when compared to the growth of control cells. Each experiment was performed at least 3 times. There was a good reproducibility between replicate wells with standard errors below 10%.

## 4. Conclusions

In the current work, we successfully prepared and developed 2,3-diamino-6-benzoyl-5-methylthieno[2,3-d]pyrimidin-4(3*H*)-one (**3**) as starting material to synthesize various new heterocyclic, such as thieno[2,3-d][1,2,4]triazolo[1,5-a] pyrimidinone-thieno[2,3-d] [1,2,4]triazolo [1,5-a]pyrimidinones, thienopyrimidotriazolothiazine-diones, pyrrolotriazolothienopyrimidines, thienopyrimidotriazolopyrroloquinoxalindiones, thienopyrimi-dotriazolopyrrolo-oxathiinoquinoxalines, 1,4-oxathiinopyrrolo-triazolothienopyrimidin- triones, imidazopyrrolotriazolothienopyrimidindiones and 1,2,4-triazoloimidazopyrrolo-triazolothienopyrimidine-diones, which were prepared and described using different methods and high techniques that produced good yields. In addition, the anticancer properties of the new compounds were studied in vitro; we in screened all compounds for cytotoxic and antiproliferative effects against the types of human carcinoma cancer cell line (**CNE2**, **KB**, **MCF-7** and **MGC-803**). The compounds **20**, **19**, **17**, **16**, **11** and **18** showed potent cytotoxic and anticancer efficacy, because these compounds contain many functional groups and many heterocyclic, such as the thienopyrimidine, 1,2,4-triazole, 1,4-thiazine, pyrrole, quinoxaline, 1,4-oxathiine and imidazole moieties, according to previous scientific studies. 1,2,4-triazolo-imidazopyrrolotriazolo-thienopyrimidin-dione (**20**) is a promising anticancer molecule with multiple modes of actions, such as antiproliferative and angiopreventive effects, together with its remarkable apoptosis-inducing action [48,49,50].

## Figures and Tables

**Table 1:** Cytotoxic activity of the new compounds against different human cancer cell lines

Compounds	In Vitro Cytotoxicity IC_50_ (µM)			
	CNE2 ^a^	KB ^a^	MCF-7 ^a^	MGC-803 ^a^
**1**	>50	>50	>50	>50
**2**	>50	>50	>50	>50
**3**	33.5 ± 1.8	32.2 ± 1.2	34.1 ± 1.3	35.7 ± 1.5
**4**	21.3 ± 1.1	20.5 ± 1.3	20.9 ± 1.2	21.5 ± 1.4
**5**	13.1 ± 1.1	12.3 ± 1.4	12.5 ± 1.3	12.8 ± 1.2
**6a**	26.3 ± 1.4	25.8 ± 1.7	26.9 ± 1.9	27.5 ± 1.3
**6b**	24.5 ± 1.5	23.1 ± 1.2	24.2 ± 1.4	25.2 ± 1.1
**6c**	25.1 ± 1.2	24.2 ± 1.3	25.5 ± 1.5	26.8 ± 1.8
**7**	14.2 ± 1.7	13.5 ± 1.5	13.7 ± 1.1	13.9 ± 1.4
**8**	29.1 ± 1.2	28.7 ± 1.1	29.5 ± 1.8	30.2 ± 1.6
**9**	18.3 ± 1.7	17.2 ± 1.5	17.8 ± 1.4	18.5 ± 1.3
**10**	19.1 ± 1.6	18.4 ± 1.2	18.7 ± 1.3	19.2 ± 1.5
**11**	12.1 ± 1.2	11.1 ± 1.3	11.8 ± 1.5	11.7 ± 1.4
**12**	16.1 ± 1.4	15.3 ± 1.3	15.9 ± 1.2	16.5 ± 1.1
**13**	15.4 ± 1.3	14.1 ± 1.2	14.8 ± 1.1	15.7 ± 1.2
**14**	20.5 ± 1.8	19.8 ± 1.5	19.8 ± 1.4	20.4 ± 1.6
**15**	12.7 ± 1.2	11.7 ± 1.6	12.1 ± 1.2	12.2 ± 1.4
**16**	11.9 ± 1.5	10.9 ± 1.2	11.6 ± 1.1	11.5 ± 1.3
**17**	11.8 ± 1.4	10.8 ± 1.1	11.5 ± 1.4	11.3 ± 1.5
**18**	12.4 ± 1.5	11.3 ± 1.4	11.9 ± 1.3	11.9 ± 1.1
**19**	11.7 ± 1.3	10.7 ± 1.5	11.4 ± 1.1	11.1 ± 1.2
**20**	11.6 ± 1.1	10.5 ± 1.5	11.3 ± 1.2	10.9 ± 1.4
**5-Fluorouracil**	11.8 ± 1.4	10.7 ± 1.2	11.5 ± 1.3	11.1 ± 1.1

^a^**CNE2** cells are drug-sensitive human nasopharyngeal carcinoma cells; **KB** cells are drug-sensitive human oral carcinoma cells; **MCF-7** cells are drug-sensitive human breast adenocarcinoma cells and **MGC-803** cells are drug-sensitive human gastric carcinoma cells.

**Table 2:** The best new compounds to cytotoxic activity against different human cancer cell lines.

Compounds/Chemical Structures	In Vitro Cytotoxicity IC_50_ (µM)			
	CNE2 ^a^	KB ^a^	MCF-7 ^a^	MGC-803 ^a^
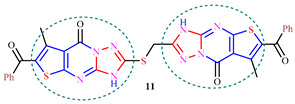	12.1 ± 1.2	11.1 ± 1.3	11.8 ± 1.5	11.7 ± 1.4
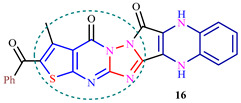	11.9 ± 1.5	10.9 ± 1.2	11.6 ± 1.1	11.5 ± 1.3
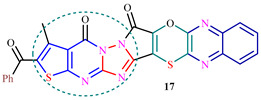	11.8 ± 1.4	10.8 ± 1.1	11.5 ± 1.4	11.3 ± 1.5
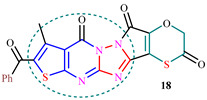	12.4 ± 1.5	11.3 ± 1.4	11.9 ± 1.3	11.9 ± 1.1
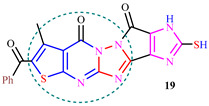	11.7 ± 1.3	10.7 ± 1.5	11.4 ± 1.1	11.1 ± 1.2
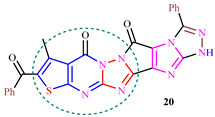	11.6 ± 1.1	10.5 ± 1.5	11.3 ± 1.2	10.9 ± 1.4
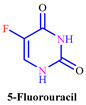	11.8 ± 1.4	10.7 ± 1.2	11.5 ± 1.3	11.1 ± 1.1

^a^**CNE2** cells are drug-sensitive human nasopharyngeal carcinoma cells; **KB** cells are drug-sensitive human oral carcinoma cells; **MCF-7** cells are drug-sensitive human breast adenocarcinoma cells and **MGC-803** cells are drug-sensitive human gastric carcinoma cells.

## Data Availability

All data are contained within the article and the experimental section.

## Sample Availability

Samples of the synthesized compounds are available from the authors.
